# Ex Vivo Effect of Novel Lipophosphonoxins on Root Canal Biofilm Produced by *Enterococcus faecalis:* Pilot Study

**DOI:** 10.3390/life12010129

**Published:** 2022-01-17

**Authors:** Yuliya Morozova, Iva Voborná, Radovan Žižka, Kateřina Bogdanová, Renata Večeřová, Dominik Rejman, Milan Kolář, Duy Dinh Do Pham, Pavel Holík, Roman Moštěk, Matej Rosa, Lenka Pospíšilová

**Affiliations:** 1 Institute of Dentistry and Oral Sciences, Faculty of Medicine and Dentistry, Palacký University, Palackého 12, 772 00 Olomouc, Czech Republic; radovan.zizka@upol.cz (R.Ž.); pavel.holik@upol.cz (P.H.); roman.mostek@upol.cz (R.M.); matej.rosa@upol.cz (M.R.); lenka.pospisilova@upol.cz (L.P.); 2 Department of Microbiology, Faculty of Medicine and Dentistry, Palacký University, Hněvotínská 3, 775 15 Olomouc, Czech Republic; katerina.bogdanova@fnol.cz (K.B.); renata.vecerova@fnol.cz (R.V.); milan.kolar@fnol.cz (M.K.); 3 Institute of Organic Chemistry and Biochemistry, Czech Academy of Sciences, Fleming Square 542/2, 160 00 Prague, Czech Republic; dominik.rejman@uochb.cas.cz (D.R.); duydinh.dopham@uochb.cas.cz (D.D.D.P.)

**Keywords:** root canal, biofilm, *E. faecalis*, sodium hypochlorite, chlorhexidine digluconate, EDTA, lipophosphonoxins

## Abstract

(1) Background: The root canal system has complex anatomical and histological features that make it impossible to completely remove all bacteria by mechanical means only; they must be supplemented with disinfectant irrigation. Current disinfectants are unable to eliminate certain microorganisms that persist in the root canal, resulting in treatment failure. At the Institute of Organic Chemistry and Biochemistry, Prague, novel substances with the bactericidal effect, termed lipophosphonoxins (LPPOs), have been discovered. The aim of this pilot study was to investigate the ex vivo effects of second- and third-generation LPPOs on *Enterococcus faecalis* and compare them with 5% sodium hypochlorite (NaOCl), 0.12% chlorhexidine digluconate, and 17% ethylenediaminetetraacetic acid (EDTA). (2) Methods: The root canal’s dentin was used as a carrier for biofilm formation in the extracted human mature mandibular premolars. The samples were filled with cultivation broth and 0.25% glucose with tested solutions. In control samples, only fresh cultivation broth (negative control) and cultivation broth with bacterial suspension (growth control) were used. Each sample was inoculated with *E. faecalis* CCM4224 except for the negative control, and cultivation was performed. To determine the number of planktonic cells, the sample content was inoculated on blood agar. To evaluate biofilm formation inhibition, samples were placed in tubes with BHI. (3) Results: LPPOs exhibited a reduction in biofilm growth and bacteria comparable to NaOCl, and they were superior to other tested disinfectants. (4) Conclusions: The study results suggest the effect of lipophosphonoxins on *E. faecalis* CCM 4224 reduces planktonic bacterial cells and inhibits formation of biofilm in root canal samples.

## 1. Introduction

The human oral cavity has a unique microbiome containing more than 1000 different bacterial species. Some of which, under certain conditions, can cause various pathological changes in hard dental tissues (tooth decay), dental pulp (reversible/irreversible pulpitis or pulp necrosis), or periapical tissues (apical periodontitis). Without appropriate treatment, some of them can lead to serious pathological changes in various organs (e.g., the cardiovascular system, kidneys, or lungs). They can also be the cause of endoprostheses colonization. Oral microorganisms have the capability to form biofilms on distinct surfaces, such as hard dental tissues and soft oral tissues. Biofilm generally refers to a mode of microbial growth, where dynamic microbial communities composed of microcolonies that contain interacting sessile bacterial cells are irreversibly attached to a solid substratum or interface, as well as to each other [1]. These microcolonies are embedded in a self-made matrix of extracellular polymeric substances and have an altered phenotype depending on their gene expression and growth rate. Bacteria contained in a biofilm are extremely resistant toward antibiotic treatment [2]. The most common bacteria of endodontic biofilm are *Enterococcus faecalis*, *Staphylococcus aureus*, *Streptococcus sanguinis*, *Streptococcus intermedius*, *Prevotella intermedia*, and *Porphyromonas endodontalis*. These bacteria can cause various pathological states requiring endodontic treatment [3].

Due to the complex anatomical and histological features of the root canal system, it is technically impossible to completely remove all present bacteria from the surfaces and inner structures by mechanical means only [4]. The root canal system has an extremely complicated anatomy, where—in addition to the the main canal, which can have different shapes, diameters, and various numbers of apical foramens—different accessory canals, as well as apical ramifications, may be present. Due to their small size and poor accessibility, it is almost impossible to mechanically shape the root canal system. Therefore, almost 50% of the root canal wall remains untreated, and bacteria can survive there [5]. This means that the mechanical treatment must be supplemented with additional support, such as chemical solutions with disinfecting properties such as irrigants.

The most frequently used irrigant in clinical endodontics is 0.5–5.25% sodium hypochlorite (NaOCl) [1]. Due to its strong antibacterial properties, it is effective against the majority of root canal bacteria. It also has proteolytic and lubricant effects, as well as the ability to dissolve organic materials. According to Clegg et al., just 6% NaOCl was able to remove artificial biofilm and kill bacteria [6]. Nevertheless, this disinfectant has its limitations and disadvantages, such as aggressive behavior to soft and periapical tissues in the case of extrusion, as well as a risk of allergic and cytotoxic reactions, foul taste and smell, ineffectiveness in smear layer removal, and the ability to bleach fabric and cause corrosion of metal objects [4,7]. Therefore, other less aggressive disinfectants, such as 0.2% chlorhexidine gluconate, iodine potassium iodide solution, MTAD (a mixture of doxycycline, citric acid, and a detergent Tween 80), or saline solution, may be used [1]. However, these disinfectants also have numerous side effects [1].

Ethylenediaminetetraacetic acid (EDTA) is another substance that is used in endodontic irrigation due to its chelating properties and ability to remove the inorganic part of the smear layer [1]. With prolonged exposure, EDTA can also remove metal ions from the bacterial cell membrane that can eventually lead to bacteria degradation [8]. Additionally, it has an antifungal effect and can detach biofilm adhesion from the root canal [9]. Generally, EDTA is used after NaOCl irrigation as part of sequential chelation.

Similar chelating properties are shown by 1-hydroxyethylidene-1,1-diphosphonic acid (HEDP). It is used as a chelating agent after NaOCl application in continual chelation [10].

Despite the use of different methods of irrigation application (manual dynamic activation with endodontic files, gutta-percha points, sonic and ultrasonic vibration, and application of negative pressure during irrigation) and activation (photodynamic therapy, photoinduced photoacoustic streaming, and laser), some microorganisms that persist in the root canal are unable to be eliminated, resulting in treatment failure [11,12]. These microorganisms are frequently *E. faecalis; Fusobacterium nucleatum; Prevotella* spp.; *Campylobacter rectus;* different species of streptococci, lactobacilli, staphylococci, and *Actinomyces* spp.; and various others [13]. One possible method to eliminate them is the application of intracanal medicaments, such as calcium hydroxide and chlorhexidine gel [4]. The most useful medicament, calcium hydroxide, has a strong antibacterial effect due to its alkaline pH (12.5–12.8) and wide range of antimicrobial activity [14]. Calcium hydroxide also has antiendotoxin activity, and it can dissolve necrotic tissue remnants. However, its effect on root canal biofilm is controversial [14]. Extrusion of calcium hydroxide into the periapical region is painful for the patient and can lead to tissue necrosis [4]. Despite its strong antimicrobial activity, some microorganisms, such as *E. faecalis* and *Candida albicans*, are able to survive in the conditions of an alkaline environment and persist in the root canal. Similar to calcium hydroxide, 2% chlorhexidine gel has a controversial influence on endodontic biofilm [14,15,16].

At the Institute of Organic Chemistry and Biochemistry, Prague, novel substances that have a bactericidal effect on both Gram-positive and -negative bacteria due to selective disruption of the cytoplasmic membrane termed lipophosphonoxins (LPPOs) have been discovered.

The advantages of LPPOs are (1) a modular structure allowing for the fine-tuning of their properties, (2) excellent chemical and biochemical stability, and (3) simplicity of synthesis.

**Figure 1:**
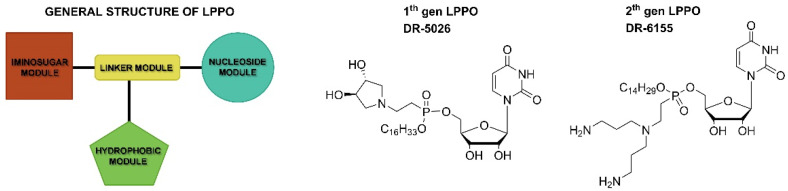
General structure of LPPO and examples of first- and second-generation LPPO.

The general structure of lipophosphonoxins consists of four modules: (i) a nucleoside module, (ii) an iminosugar module, (iii) a hydrophobic module (a lipophilic alkyl chain), and (iv) a phosphonate linker module that holds together modules (i)–(iii) (Figure 1).

First-generation LPPOs were bactericidal against various Gram-positive species, including multiresistant strains, such as vancomycin-resistant *E. faecium* and methicillin-resistant *S. aureus*. The minimum inhibitory concentration values of the best inhibitors were in the 1–12 mg/L range, while their cytotoxic concentrations against human cell lines were significantly above this range [17,18,19]. Based on a study of the mechanism of action for the first generation, the second generation of LPPOs was designed and synthesized, providing compounds with a broadened spectrum of activity against several clinically relevant Gram-negative bacteria (*Escherichia coli* and *Pseudomonas aeruginosa*) [18,20]. Currently, the third and fourth generations of LPPOs are under development. They seem to outperform both first- and second-generation LPPOs in terms of antibacterial activity and selectivity. LPPOs of the first, second, and third generations do not irritate skin, and since they are not absorbed into the GIT, they are not toxic at peroral application (MTD for p.o. administration in mice of second generation LPPO is up to 2000 mg/kg of body weight) [20], which makes them ideal for use in the treatment of endodontic infections.

The aim of this study was to compare the *E. faecalis* eradication ability of selected LPPOs from the second (DR-6328) and third (DR-6487) generations with the most commonly used endodontic disinfectants: 2.5% aqueous sodium hypochlorite (NaOCl), 0.12% chlorhexidine digluconate, and 17% EDTA.

## 2. Materials and Methods

### 2.1. Teeth Preparation

**Figure 2:**
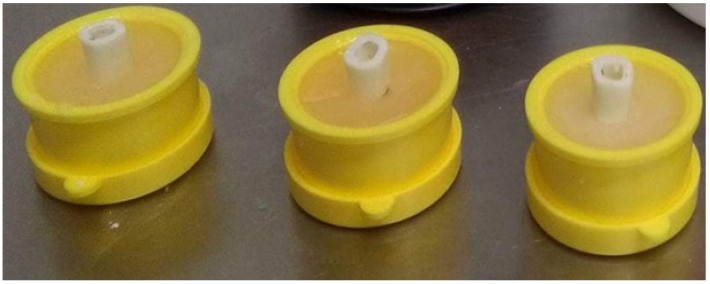
The root samples fixed in rubber holders.

To simulate the endodontic infection, the root canal system’s dentin was used as a carrier for biofilm formation in the extracted human mature mandibular premolars (total number is 15). The study was conducted according to the guidelines of the Declaration of Helsinki. Informed consent of patients whose teeth were used in the study was obtained, as well as approval of the Ethical Committee of Faculty Hospital and Palacký University in Olomouc (NV-19-05-00192). Immediately after extraction, the teeth were placed into 5% NaOCl (Parcan, Septodont, Paris, France) for 30 min to remove organic tissues and were subsequently disinfected in a 10% formalin solution for 1 week. Afterward, the teeth were removed and rinsed with distilled water, and periapical radiographs were captured. The clinical crown was removed at or near the cement–enamel junction using a diamond burr (Dentsply Sirona, York, PA, USA) in a high-speed handpiece (KaVo, Berlin, Germany) with water cooling, and roots with a standard length of 15 mm were obtained. A 3 mm reservoir in the coronal third of the root was prepared using Gates Glidden drills (#2–4) (Dentsply Mailefer, Ballaigues, Switzerland) [3,21]. The root canals were shaped by a ProTaper Universal system (S1–F5) (Dentsply Mailefer, Ballaigues, Switzerland), using the crown down preparation technique for length 1 mm shorter than apex, maintaining patency with a K-file ISO 25 (Dentsply Mailefer, Ballaigues, Switzerland). During shaping, the root canals were irrigated with 2 mL of 1% NaOCl after every change in instrument, with a final irrigation of 17% solution of EDTA (Meta Biomed, Cheongju, Korea) for 1 min to remove the smear layer. Finally, the teeth were fixed by resin in a rubber holder and autoclaved at 121 °C and 98.6 kPa for 2 h (Figure 2). Root canal samples were then rinsed with saline and immersed in artificial saliva (Xerostom, Biocosmetics Laboratories, Madrid, Spain).

### 2.2. Lipophosphonoxins Preparation

Compound DR-6328 was prepared according to published procedure [20]. The compound is coded as 8b in this article. Compound DR-6487 was prepared using a similar procedure; the detailed synthesis procedure will be published elsewhere.

### 2.3. Determination of Minimum Inhibitory Concentrations (MICs) and Minimum Bactericidal Concentrations (MBCs) of Tested LPPOs and Endodontic Disinfectants

**Figure 3:**
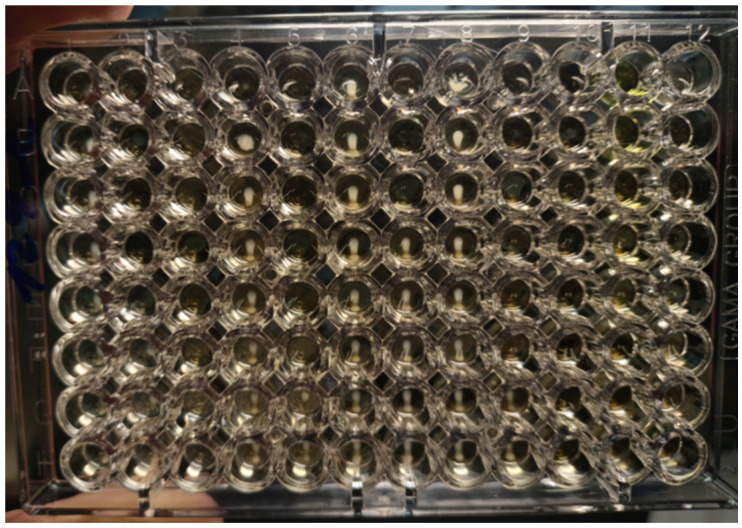
Microtiter plates filled with Mueller–Hinton growth medium and inoculated with *E. faecalis* CCM 4224 (after incubation for 18 ± 2 h at 35 ± 1 °C). In each column is a single antibiotic (a total of 12 antibiotics for one plate) that is diluted in eight wells. MIC is defined as the lowest concentration of antibiotic inhibiting the growth of tested strain. MIC is compared with breakpoints (criterion for determination of resistance or susceptibility defined by EUCAST. If MIC ≤ breakpoint, the microbe is susceptible; if MIC > breakpoint, the microbe is resistant.

**Figure 4:**
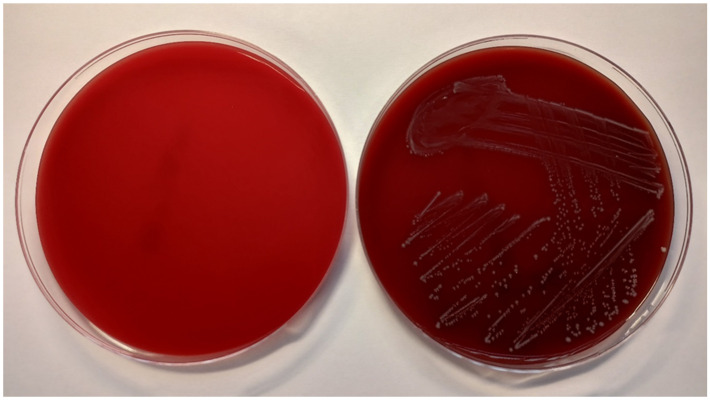
Columbia blood agar without bacterial culture (**left**) and inoculated with *E. faecalis* CCM 4224 (**right**) after 18 ± 2 h of incubation at 35 ± 1 °C.

The antimicrobial activity of the tested solutions was tested in accordance with the guidelines from the European Committee on Antimicrobial Susceptibility Testing (EUCAST) [22]. The minimum inhibitory concentration (MIC) required for inhibition of bacterial growth was determined by the microdilution method, and the minimum bactericidal concentration (MBC, a minimum concentration required for irreversible inhibition, i.e., killing the bacterium after a defined period of incubation) was determined by the inoculation method on agar (Figure 3). LPPO samples were diluted exponentially and tested in microtiter plates. Mueller–Hinton broth (MH; Bio-Rad, Prague, Czech Republic) with adjusted cations was used as the culture medium. Then, the plates were inoculated with a standard amount of the tested microbe. The inoculum density in each well was equal to 10^6^ CFU/mL. The MIC was determined after 24 h of incubation at 35 °C as the lowest concentration of the tested substance that inhibited the growth of the bacterial strain. To determine minimum bactericidal concentrations (MBCs), the contents of the wells with visibly inhibited growth were inoculated onto blood agar (TRIOS, Prague, Czech Republic) and incubated for an additional 24 h at 35 °C (Figure 4). Negative growth of microbial colonies was used to determine the MBCs. The MICs and MBCs of tested substances are shown in Table 1.

### 2.4. Phenotypic Confirmation of Biofilm Production

For this study, *E. faecalis* CCM 4224 was tested for biofilm production in cultivation broth supplemented with 0.25% glucose by the Christensen method modified by Stepanovic et al. [23,24]. Following incubation in microtiter plates, the wells were rinsed out to remove planktonic cells, and the formed biofilm was fixed with methanol and stained with crystal violet. The stains from the fixed biofilm were washed out by adding acetic acid and absorbance at 570 nm was measured (Dynex MRX spectrophotometer, Dynex Technologies, Prague, Czech Republic), as well as negative control (N-wells without bacterial strains that underwent the same process described above). The positive/negative production of biofilm was estimated using the following relations:

≤2 x (NC + 3 x SD NC)—negative biofilm production

≥4 x (NC + 3 x SD NC)—positive biofilm production

where NC is the negative control’s absorbance and SD NC is the negative control’s standard deviation obtained from four measurements.

### 2.5. Determination of Biofilm Growth Inhibition by Tested Substances

For the microbiological experiments on biofilm growth inhibition, 10x MBC and 50x MBC of DR-6328, DR-6487, chlorhexidine digluconate, and EDTA were used. NaOCl was used only in one concentration: 17x MBC (2.5%; this concentration is the most frequently used concentration of NaOCl in clinical practice).

Root canal samples were filled with cultivation broth (Brain Heart Infusion, HiMedia) and supplied with 0.25% glucose for the tested LPPOs, chlorhexidine digluconate, EDTA (all in concentrations of 10x and 50x MBC), and 2.5% NaOCl. In the control root canal samples, only fresh cultivation broth (negative control) and cultivation broth with bacterial suspension (growth control) were used. Each sample was inoculated with *E. faecalis* CCM 4224 (5 µL of 1 × 10^6^ CFU/mL), except for the negative control. The cultivation was carried out at 35 °C for 24 h. To determine the number of planktonic cells, 10 µL of sample content was directly inoculated on blood agar (TRIOS). To evaluate biofilm formation inhibition of tested substances, teeth samples were rinsed with saline and placed in test tubes with BHI, sonicated in water bath at 160 W for 2 min (Sonorex RK 156, Fischer Scientific, Waltham, MA, USA), and thoroughly shaken. A total of 10 µL of medium with released biofilm was inoculated on blood agar. After cultivation of blood agar at 35 °C for 24 h, the semiquantitative evaluation of bacterial growth was performed (detection limit 10^2^ CFU/mL). All experiments were performed in duplicate.

## 3. Results

**Figure 5:**
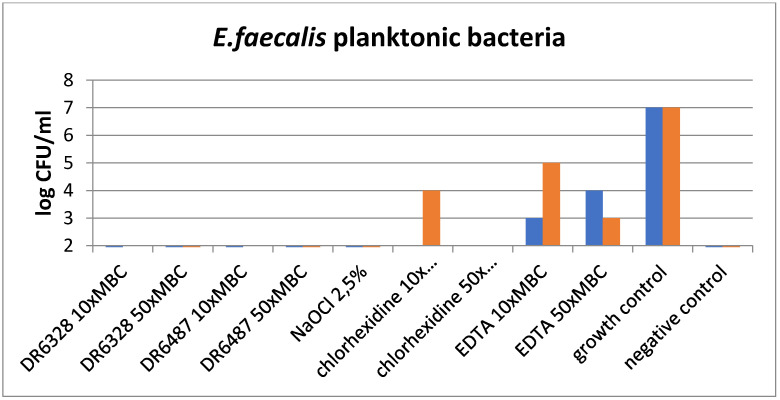
Effect of the tested substances on planktonic bacteria (testing in duplicate).

**Figure 6:**
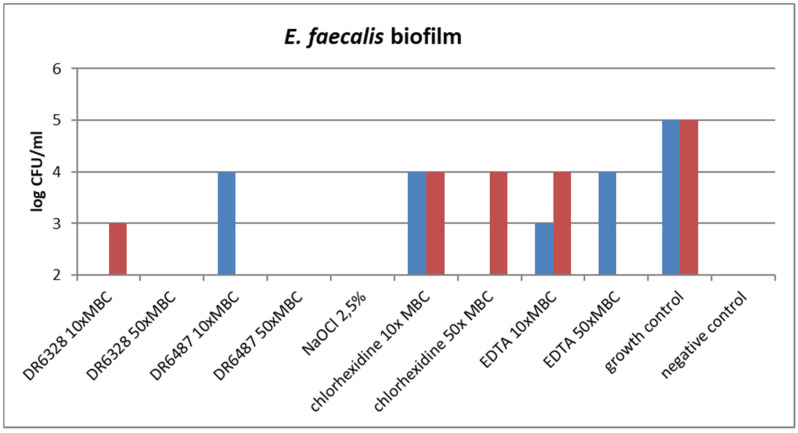
Effect of the tested substances on biofilm growth inhibition (testing in duplicate).

The results of the experiments are shown in Figure 5 and Figure 6. Figure 5 graphs the effect of the tested substances on the planktonic bacteria *E. faecalis* and demonstrates the visible reduction in bacteria with the usage of LPPOs (with both types of LPPOs at each concentration) compared to the growth control, as well as chlorhexidine and EDTA. A similar effect was demonstrated for NaOCl.

The ability of the tested substances to suppress biofilm formation is shown in Figure 6. A significant reduction in biofilm growth was detected for NaOCl and for both tested LPPOs (primarily in the higher concentration tested) in comparison to the other tested endodontic disinfectants, chlorhexidine and EDTA.

Due to the insufficient number of teeth samples, statistical analysis has not been performed.

## 4. Discussion

In this pilot in vitro study, we aimed to compare the efficacy of three standard and two novel disinfectants of the root canal system; *E. faecalis* was chosen as the investigated microorganism for its in vivo resistance to current endodontic disinfectants. This bacterium possesses virulence factors, such as aggregation substance, enterococcal surface protein, gelatinase, cytolysin toxin, extracellular superoxide formation, capsular polysaccharides, and antibiotic resistance determinant [21,25], which enable its survival under harsh conditions. In addition, the bacterium is able to withstand alkaline pH up to 11.5, which can be found in a few intracanal medicaments, such as calcium hydroxide [25,26]. Due to these properties, *E. faecalis* is considered a main reason of root canal treatment failure [25,27]. Therefore, one of the main directions of modern endodontic research is to develop novel disinfectants that are effective against *E. faecalis*.

Our pilot in vitro study confirmed the efficacy of 2.5% NaOCl and 0.12% chlorhexidine digluconate against *E. faecalis* planktonic bacteria and biofilm growth. These results fully agree with other studies investigating the efficiency of these root canal disinfectants. Sodium hypochlorite is a strong oxidant that releases chlorine [28,29,30]. It is highly hypertonic and strongly alkali (pH 11 to 13). It can dissolve vital and necrotic dental pulp residues and eliminate Gram-positive and -negative bacteria, viruses, and fungi [29,30,31,32,33,34]. In the literature and different irrigation protocols for clinical application, the recommendations for NaOCl use in different concentrations can be found [35]. Shehab et al. reported the bactericidal effect of different concentrations (0.5%, 2.5%, and 5.25%) of a NaOCl solution against *E. faecalis* in root canal samples applied at two selected times (2 and 5 min) for each concentration [36]. Significantly better results were demonstrated for 2.5% (2 min application) and 5.25% (2 and 5 min application). This finding could be explained by the fact that a longer application time increases the efficiency of sodium hypochlorite. Similar findings were reported by Gomes et al. [7]. It is clear that higher concentrations of NaOCl should have a higher antimicrobial effect. Nevertheless, regarding NaOCl, the antimicrobial effect for concentrations of 1% and higher does not change. Higher concentrations have a better proteolytic effect that is important for the dissolution of organic remnants of dental pulp inside the root canal due to the effect of hypochlorous acid released by the saponification reaction [28,37]. Moreover, higher concentrations of NaOCl have more substantial adverse effects, primarily periapical and soft tissue necrosis formation in the case of accidental extrusion [31,38,39].

In our study, we tested the most common concentration of sodium hypochlorite that is used in clinical practice: 2.5%. We detected its better ability to reduce *E. faecalis* planktonic bacteria and biofilm growth compared to chlorhexidine digluconate. Chlorhexidine is a cationic biguanide that is able to connect to microorganism cell walls and can lead to the destruction of components [40]. It is effective against both Gram-positive and -negative bacteria [15]. Similar to root canal disinfectant, it is used at concentrations of 0.02–2%. The most common form is a solution or gel. It is safer than NaOCl, and it is not aggressive to periapical and soft tissue [30]. This property enables the usage of chlorhexidine in a way similar to the most frequent antimicrobial substances in periodontology: mouthwash. It has cytotoxicity to human cells and can lead to staining of the surface of the teeth, reconstructions, and tongue. Additionally, frequent use can cause alteration in taste [16]. However, these adverse effects are more common if the chlorhexidine is used as a mouthwash. With respect to root canal irrigation, it is necessary to avoid mixing chlorhexidine and NaOCl during their applications because of their reaction: parachloroaniline is formed as a carcinogenic substance with brown color precipitated into the root canal walls [41,42,43].

If chlorhexidine is used properly, it is able to reduce the most common microorganism in the root canal system. In their in vitro study, Gomes et al. detected the bactericidal effect of 0.02–2% chlorhexidine against *E. faecalis* cells in 30 s or less [7]. Sena et al. demonstrated the bactericidal effect of 2% liquid chlorhexidine on single-species biofilms formed by *E. faecalis*, *S. aureus*, *C. albicans*, *P. intermedia*, *Porphyromonas gingivalis*, *P. endodontalis*, and *F. nucleatum* biofilm [44]. Vianna et al. tested in vitro the efficacy of the combination of sodium hypochlorite (1%, 2.5%, and 5.25%) and 2% chlorhexidine in both gel and liquid forms against *E. faecalis* compared to the antimicrobial activity of the same irrigants. They found better effectivity of NaOCl 1% and 2.5%. Chlorhexidine gel was less effective against *E. faecalis*. The authors also discovered that the combination of NaOCl and chlorhexidine did not have better antimicrobial activity than chlorhexidine applied alone [45]. Furthermore, in clinical application, it is necessary to avoid mixing chlorhexidine and NaOCl during their applications, as it creates a carcinogenic substance with brown color that precipitates into the root canal walls [41,42,43].

The next irrigant that was used in our study was 17% EDTA. This substance is mainly used in endodontics after NaOCl as the final irrigant due to its chelating properties that bind inorganic substances from the smear layer in the root canal dentinal walls, resulting in dissolution of the smear layer and improvement in sealer adhesion. It has little or no antimicrobial effect, but a few studies have shown the antifungal effect of EDTA [46]. In our study, we detected some antibacterial activity in 17% EDTA, but it was markedly lower than that of the other tested irrigants.

Due to previously mentioned disadvantages of currently available endodontic irrigants, the search for novel effective and safe disinfectants is ongoing. In our study, we investigated the novel lipophosphonoxins that have been developed at the Institute of Organic Chemistry and Biochemistry (Prague, Czech Republic). They are small amphiphilic molecules bearing positive charge(s). LPPOs of the first generation demonstrated excellent bactericidal activity against various Gram-positive species, including multiresistant strains (vancomycin-resistant enterococci and methicillin-resistant *S. aureus*) [17,19,47]. However, LPPOs of the first generation are ineffective against Gram-negative bacteria. By redesigning the iminosugar module, the LPPOs of the second generation were developed [47]. They demonstrated increased efficacy against Gram-positive species, as well as antibacterial activity against serious Gram-negative pathogens (clinically relevant strains of *E. coli*, *P. aeruginosa*, and *Salmonella enterica* subsp. Enteritidis [20]. Another advantage of LPPOs is their modular structure and simple, straightforward synthesis that allows for fine-tuning of their properties in systematic matter via structural alteration.

Zborníková et al. performed an in vitro evaluation of LPPOs of the second generation as an antibacterial additive to bone cement that was applied on the orthopedic implant surface to prevent microbial biofilm formation [47]. The authors demonstrated excellent thermostability of the tested LPPOs, unchanged tensile strength, elongation of the break properties in the cements containing LPPOs, convenient elution kinetics, as well as the strong antibiofilm activity of the LPPOs of the second generation cements, including bacterial resistance to antibiotic gentamicin. Therefore, LPPOs show potential as antimicrobial additives to bone cements.

Other studies investigating LPPO substances noted their potential for development as antibacterial agents for local application [17].

In our study, LPPOs were effective against the tested bacteria with comparable activity to 2.5% NaOCl. Since LPPOs are free of NaOCl’s adverse effects (aggressive behavior to soft and periapical tissues in the case of extrusion, as well as risk of allergic and cytotoxic reactions, damage to eyes or skin in the case of accidental application, foul taste and smell, ineffectiveness in smear layer removal, and the ability to bleach fabric and cause corrosion of metal objects), they show promise as new disinfectants for endodontics [17,18,19,20]. However, further robust investigations on larger samples with higher numbers of structurally diverse lipophosphonoxins, including the latest third and fourth generations of LPPOs, and other bacterial species, with proper statistical analysis, are necessary. We also plan to implement investigation on animal models to evaluate the LPPOs’ possible adverse effect on periapical and oral soft tissues.

## 5. Conclusions

In our pilot ex vivo study, we established the effect of novel bactericidal lipophosphonoxins on the strain *E. faecalis* CCM 4224 to reduce the planktonic bacterial cells, as well as its ability to inhibit biofilm formation in root canal samples. Compared to common endodontic disinfectants, the effect of LPPOs is similar (in the case of NaOCl) or better (in the cases of chlorhexidine digluconate and EDTA). LPPOs are promising substances for dentistry. However, further testing of their effects on an extended set of bacterial species is needed.

## Figures and Tables

**Table 1:** Minimum inhibitory concentration and minimum bactericidal concentration values of the tested substances.

Tested Substance	MIC	MBC
LPPO 2nd gen. (DR-6328)	0.0008%/8 mg/L	0.0016%/16 mg/L
LPPO 3rd gen. (DR-6487)	0.0004%/4 mg/L	0.0004%/4 mg/L
NaOCl	0.08%/800 mg/L	0.15%/1.5 g/L
Chlorhexidine digluconate	0.0002%/2 mg/L	0.0002%/2 mg/L
EDTA	0.0085%/85 mg/L	0.0085%/85 mg/L
